# Striatal Cholinergic Interneurons Modulate Spike-Timing in Striosomes and Matrix by an Amphetamine-Sensitive Mechanism

**DOI:** 10.3389/fnana.2017.00020

**Published:** 2017-03-21

**Authors:** Jill R. Crittenden, Carolyn J. Lacey, Feng-Ju Weng, Catherine E. Garrison, Daniel J. Gibson, Yingxi Lin, Ann M. Graybiel

**Affiliations:** Department of Brain and Cognitive Sciences, McGovern Institute for Brain Research, Massachusetts Institute of TechnologyCambridge, MA, USA

**Keywords:** striosome, striatum, amphetamine, stereotypy, cholinergic interneuron

## Abstract

The striatum is key for action-selection and the motivation to move. Dopamine and acetylcholine release sites are enriched in the striatum and are cross-regulated, possibly to achieve optimal behavior. Drugs of abuse, which promote abnormally high dopamine release, disrupt normal action-selection and drive restricted, repetitive behaviors (stereotypies). Stereotypies occur in a variety of disorders including obsessive-compulsive disorder, autism, schizophrenia and Huntington's disease, as well as in addictive states. The severity of drug-induced stereotypy is correlated with induction of c-Fos expression in striosomes, a striatal compartment that is related to the limbic system and that directly projects to dopamine-producing neurons of the substantia nigra. These characteristics of striosomes contrast with the properties of the extra-striosomal matrix, which has strong sensorimotor and associative circuit inputs and outputs. Disruption of acetylcholine signaling in the striatum blocks the striosome-predominant c-Fos expression pattern induced by drugs of abuse and alters drug-induced stereotypy. The activity of striatal cholinergic interneurons is associated with behaviors related to sensory cues, and cortical inputs to striosomes can bias action-selection in the face of conflicting cues. The neurons and neuropil of striosomes and matrix neurons have observably separate distributions, both at the input level in the striatum and at the output level in the substantia nigra. Notably, cholinergic axons readily cross compartment borders, providing a potential route for local cross-compartment communication to maintain a balance between striosomal and matrix activity. We show here, by slice electrophysiology in transgenic mice, that repetitive evoked firing patterns in striosomal and matrix striatal projection neurons (SPNs) are interrupted by optogenetic activation of cholinergic interneurons either by the addition or the deletion of spikes. We demonstrate that this cholinergic modulation of projection neurons is blocked in brain slices taken from mice exposed to amphetamine and engaged in amphetamine-induced stereotypy, and lacking responsiveness to salient cues. Our findings support a model whereby activity in striosomes is normally under strong regulation by cholinergic interneurons, favoring behavioral flexibility, but that in animals with drug-induced stereotypy, this cholinergic signaling breaks down, resulting in differential modulation of striosomal activity and an inability to bias action-selection according to relevant sensory cues.

## Introduction

Pathologically repetitive and restricted behaviors, known as stereotypies, occur in numerous disorders and are notably difficult to disrupt, suggesting a failure in attention to environmental cues that might normally direct a change in behavior. Stereotypies can also be induced in humans and other animals by high doses of habit-forming drugs such as cocaine and amphetamine. Animals with drug-induced stereotypic behavior exhibit high c-Fos immediate-early gene induction in striatal projection neurons (SPNs) within the striosome compartment of the striatum, relative to low activation in SPNs located in the surrounding matrix compartment (Canales and Graybiel, 2000; Tan et al., 2000; Saka et al., 2004; Crittenden and Graybiel, 2011, 2016; Horner et al., 2012; Jedynak et al., 2012). Such differential c-Fos induction in striosomes, relative to c-Fos induction in the matrix, is blocked by intrastriatal ablation of cholinergic interneurons (referred to herein as ChIs), along with somatostatin-expressing interneurons (Saka et al., 2002), and manipulations of ChI signaling have a direct impact on the severity of cocaine- and amphetamine-induced stereotypies (Schoffelmeer et al., 2002; Collins and Izenwasser, 2004; Thomsen et al., 2010; Aliane et al., 2011; Crittenden et al., 2014). High-dose drug treatments also are related to an imbalance in activity of cortical regions that differentially project to the striatal striosome or matrix compartment (Gerfen, 1984; Kincaid and Wilson, 1996; Aliane et al., 2009; Friedman et al., 2015; Kupferschmidt et al., 2015). All together, these findings suggest that a balance in activity between striosome-based and matrix-based cortico-basal ganglia loops could be critical for behavioral flexibility.

In contrast to the widespread interconnections that occur among the D1- and D2-dopamine receptor-positive SPNs of the direct and indirect output pathways (Taverna et al., 2008), SPNs that lie in the striosome or matrix compartments seem particularly separated, not only physically but also electrophysiologically (Kawaguchi et al., 1989; Banghart et al., 2015; Lopez-Huerta et al., 2016), though with some cross-compartment crossing of their processes (Bolam et al., 1988; Walker et al., 1993). Striosomal SPNs give rise to a unique striatonigral connection to subgroups of neurons in the substantia nigra pars compacta that produce dopamine (Gerfen, 1985; Jimenez-Castellanos and Graybiel, 1989; Fujiyama et al., 2011; Watabe-Uchida et al., 2012; Crittenden et al., 2016), a neurotransmitter that is released by all known drugs of abuse and is a key driver of motivated behaviors and movement initiation (Schultz, 2011; Dodson et al., 2016; Howe and Dombeck, 2016). Further, the striosome-targeted dopamine-positive neurons project back both to striosomes and matrix, suggesting a circuit by which striosomes could exert both localized and global control over the dorsal striatum (Crittenden et al., 2016). For technical reasons, few studies have addressed how striosomal neurons are regulated, and striosomes are difficult targets because they comprise only ~15–20% of the SPN populations, which primarily occupy the extrastriosomal matrix that houses the cells of origin of the classic direct and indirect basal ganglia pathways for motor control. Yet striosomes are preferential targets of some cortical regions related to the limbic system, and they target lateral habenula-linked circuits and the dopamine-containing nigral pars compacta, both implicated in motivation and reinforcement-driven behaviors. Thus, striosomal SPNs, insofar as they act through their main GABAergic neurotransmitter mechanism, are well placed to counterbalance hyper-activation of the dopamine system.

The relative segregation of striosomal and matrix SPNs raises the question of what mechanisms maintain the normal balance of the striatal compartments, including the balanced expression of immediate-early gene products, such as c-Fos, expression that becomes striosome-predominant in animals exhibiting drug-induced stereotypy. ChIs and other interneurons that are located along the striosomal borders are leading candidates for coordinating activity between the two compartments (Graybiel et al., 1986; Sandell et al., 1986; Kubota and Kawaguchi, 1993; Aosaki et al., 1995; Mounir and Parent, 2002; Miura et al., 2007; Brimblecombe and Cragg, 2015). ChIs are thought to correspond to the tonically active neurons of the striatum (TANs), which respond to salient or instructive stimuli with an activation, pause and rebound pattern of activity (Apicella et al., 1991; Aosaki et al., 1995; Blazquez et al., 2002; Minamimoto and Kimura, 2002; Goldberg and Reynolds, 2011; Doig et al., 2014; Thorn and Graybiel, 2014). This cue-response is semi-synchronized among TANs across the striatum and is driven by input from the neocortex and from the intralaminar nuclei of the thalamus, which are themselves activated by sensory inputs (Aosaki et al., 1995; Matsumoto et al., 2001; Morris et al., 2004; Brown et al., 2010; Doig et al., 2014). Strategic switching behaviors exhibited in learning tasks are impacted by manipulations of ChIs in the striatum (Minamimoto et al., 2005; McCool et al., 2008; Brown et al., 2010; Okada et al., 2014). The extreme loss of behavioral flexibility that occurs in animals engaged in drug-induced stereotypies is likewise modulated by cholinergic manipulations (Schoffelmeer et al., 2002; Collins and Izenwasser, 2004; Thomsen et al., 2010; Aliane et al., 2011; Crittenden et al., 2014). These findings suggest that thalamic and cortical input to the ChIs of the dorsal striatum helps to re-set motor systems to modulate behavioral flexibility in the face of conflicting cues or drug-induced overload of the dopamine system (Ding et al., 2010; Minamimoto et al., 2014; Eskow Jaunarajs et al., 2015). Whether striosomal and matrix SPNs are differentially controlled by ChIs, however, has not been tested. We now have performed such a test, and have specifically asked whether we could estimate the ChIs modulation of SPN firing activity related to amphetamine-induced stereotypies by examining SPN responses in slices prepared from animals actively engaged in such behaviors.

It is known from much work on striatal slices that activation of ChIs induces GABA release from local sources to drive inhibitory responses in SPNs (Miura et al., 2006; English et al., 2012; Luo et al., 2013; Nelson et al., 2014b; Faust et al., 2016). These measurements have been made without identification of striatal compartments, but likely were made mostly in the large matrix compartment. Inoue and colleagues (Inoue et al., 2016) have now identified striosomes in a brain slice recording preparation by using young transgenic mice expressing green fluorescent protein (GFP) under the control of the tyrosine hydroxylase promoter (Matsushita et al., 2002), which is striosome-enriched in young mice (<28 days of age). Paired neuronal recordings in these mice demonstrated that SPNs, and other ChIs, can inhibit ChIs within and across compartments (Inoue et al., 2016), consistent with the fact that ChI neuropil crosses compartment borders (Graybiel et al., 1986; Crittenden et al., 2014). Whether ChI activation induces the prominent pause in activity in both striosomal and matrix SPNs was not examined.

We performed slice recordings and optogenetic stimulation of ChIs in double transgenic mice that have matrix-enriched GFP and channelrhodopsin (ChR2) in ChIs. Wide-field application of a blue light pulse to the slices induced a single spike in the ChIs. We show here that this ChI stimulation produced a brief inhibition of evoked, repetitive firing in both striosomal and matrix SPNs. We also observed, in SPNs of both the matrix and striosomes, that the optogenetic activation of ChIs occasionally induced a spike-advance in the SPN firing pattern. All of these effects were blocked by the nicotinic acetylcholine receptor antagonist, dihydro-β-erythroidine (DHβE). Furthermore, in brain slices taken from drug-treated transgenic mice engaged in amphetamine-induced stereotypies and non-respondent to salient cues, ChI stimulation failed to disrupt the evoked repetitive striosomal and matrix SPN firing. These results raise the proposal, favored here, that the loss of behavioral flexibility that occurs in animals under the influence of psychomotor stimulants could result from a failure in the ability of ChIs to reset ongoing striatal activity and motor behavior in response to relevant environmental cues.

## Materials and methods

### Mice

All procedures were approved by the Committee on Animal Care at the Massachusetts Institute of Technology, which is AAALAC accredited. Table 1 summarizes mouse lines used. Double transgenic mice were generated from intercrosses of single hemizygous mice so that no offspring were homozygous for a transgene. ChAT-IRES-Cre knock-in mice (JAX stock #006410) (Rossi et al., 2011) were on a mixed 129S6, C57Bl/6J genetic background. Mice with the ChAT-ChR2-EYFP bacterial artificial chromosome (BAC) (Zhao et al., 2011) were on a C57Bl/6 genetic background. CalDAG-GEFI (aka RasGRP2)-EGFP BAC mice (Gong et al., 2003) were on a FVB/N-Swiss Webster hybrid background. For neuroanatomical studies, ChAT-IRES-Cre knock-in mice were crossed to Ai32(RCL-ChR2(H134R)/EYFP) knock-in mice (JAX stock #024109) (Madisen et al., 2012) on a C57Bl/6 background. Experimental mice were male, 2–6 months of age, and housed under a standard light-dark cycle (lights on at 7 am and off at 7 pm), with free access to food and water. Mice with intracerebral viral injections were single-housed post-surgery, and mice that were D-amphetamine-treated were single-housed. Otherwise mice were group-housed with brothers.

**Table 1:** **Transgenic mice used in this study**.

**Transgenic mouse line**	**Description**	**References**
Single transgenic CalDAG-GEFI-GFP BAC	EGFP is inserted in the CalDAG-GEFI (aka RasGRP2) BAC and is expressed in matrix SPNs.	Gong et al., 2003; Crittenden et al., 2016
Double knock-in transgenic ChAT-Cre;Ai32	Cre is inserted in the choline acetyl transferase (ChAT) gene locus and is expressed in ChIs. Cre-dependent EYFP is inserted in the Rosa 26 locus to permit expression in most cell types that express Cre.	Rossi et al., 2011; Madisen et al., 2012
Double transgenic CalDAG-GEFI-GFP BAC;ChAT-Cre knock-in	EGFP is expressed in matrix SPNs. ChR2 is expressed in ChIs by intracerebral injection of a viral vector for Cre-dependent ChR2.	Gong et al., 2003; Rossi et al., 2011
Double BAC transgenic CalDAG-GEFI-GFP;ChATChR2	EGFP is expressed in matrix SPNs. ChR2 is inserted in a BAC containing the ChAT gene and is expressed in ChIs.	Zhao et al., 2011

### Tissue preparation, immunolabeling, and microscopy

Mice were anesthetized with Euthasol (Virbac AH Inc.; pentobarbital sodium and phenytoin sodium) and then trans-cardially perfused with 0.9% saline, followed by 4% paraformaldehyde in 0.1 M NaKPO_4_ buffer solution (PBS). Brains were then dissected, post-fixed for 90 min, stored in 20% glycerin sinking solution overnight or longer, and cut into transverse sections at 30 μm on a freezing microtome. Sections were stored in 0.1% sodium azide in 0.1 M PBS until use. Free-floating sections were rinsed in 0.01 M PBS with 0.2% Triton X-100 and then were blocked in TSA Blocking Reagent (Perkin Elmer) prior to incubation for 1 or 2 nights with immunosera against GFP (Aves Lab, GFP-1020, 1:500 dilution), yellow fluorescent protein (Abcam, Ab6556, 1:2,000 dilution), mu-opioid receptor subtype 1 (MOR1) (Abcam, Ab134054, 1:500 dilution), CalDAG-GEFI (#3752, 1:5,000, dilution) (Crittenden et al., 2004), c-Fos (Santa Cruz, sc-52, 1:10,000 dilution), or vesicular acetylcholine transferase (VAChT) (Millipore, AB1588, 1:100) followed by the appropriate secondary antisera coupled to ALEXA fluorophores (ThermoFisher Scientific, 1:300). Following secondary incubation, sections were rinsed in 0.1 M PBS, mounted on subbed glass slides and coverslipped with ProLong antifade reagent (Thermo Fisher Scientific). Imaging was performed with a Zeiss LSM 510 confocal microscope with Zen Software and the data were processed and analyzed with Fiji software (Schindelin et al., 2012).

### Amphetamine treatments and behavioral tests

Mice were injected at ~10 am in their home cage for 3 days with saline only (10 ml/kg/day, i.p.) followed by 7 daily injections of D-amphetamine (Sigma-Aldrich) dissolved in saline (7 mg/kg/day, 10 ml/kg, i.p.), or saline vehicle, followed by 7 days without treatment and then a final challenge with amphetamine (7 mg/kg) or saline. To measure behavior on challenge day, the bedding (nestlet) was removed from the cages of the test mice, the mice received their challenge injection of D-amphetamine (or saline for the control mice) and, 30 min later, a ball of bedding was introduced from a mixture from 10 cages of female mice. Saline-treated mice were challenged again with saline 7 days later and the same test was done, but with bedding re-introduced from their own home cage. Mice were videotaped and their behavior was scored for a 2 min period beginning at 5 min after the introduction of the cage bedding. An experimenter blinded to mouse treatment scored the videotapes using a keyboard scoring system with the public domain software JWatcher™, version 1.0 (University of California, Los Angeles, CA, USA, and Macquarie University, Sidney, Australia, http://www.jwatcher.ucla.edu/). Individual keys were assigned to score resting, locomotion, sniffing or touching bedding, and highly confined stereotypy. The interaction of saline-treated mice with female cage bedding vs. their own home cage bedding was compared by a paired-sample, 2-tailed Student's *t*-tests. The interaction of amphetamine-treated mice vs. saline-treated mice with female cage bedding was compared by an unpaired, 2-tailed Student's *t*-test. Statistical analysis of amphetamine-induced stereotypy and interaction with female bedding was made on mice that carry the ChAT-Cre transgene alone, but equivalent severe stereotypy was observed in all double transgenic CalDAG-GEFI-GFP;ChAT-Cre knock-in mice that were used for recording and c-Fos induction experiments.

### Intrastriatal viral injections

ChAT-IRES-Cre mice or double transgenic, CalDAG-GEFI-EGFP-BAC;ChAT-IRES-Cre mice and CalDAG-GEFI-EGFP-BAC;ChAT-ChR2-EYFP BAC mice were maintained in deep anesthesia with a continuous flow of 2% isoflurane (Southmedic Inc.) in an oxygen mixture, delivered by a nose-cone attached to a stereotaxic frame. Mice were given bilateral intrastriatal injections, via NanoFil microsyringe (World Precision Instruments), of AAV5 encoding EF1αDIO hChR2(H134R)-mCherry (University of N. Carolina vector core), 0.75 μl per site, at the following stereotactic coordinates (AP = 0.9 mm, ML = −1.9 mm and +1.9 mm, DV = 2.0 mm and 2.7 mm, relative to bregma). Mice were allowed to fully recover from surgery for 2 weeks prior to the initiation of saline or D-amphetamine injections.

### *In vitro* slice preparation and patch-clamp electrophysiology recordings

For patch-clamp recording experiments, double transgenic CalDAG-GEFI-GFP BAC;ChAT-Cre knock-in mice and CalDAG-GEFI-GFP BAC;ChAT-ChR2-EYFP BAC mice were given saline or drug treatments beginning at ~2 weeks after intrastriatal viral injection. At 30 min post-injection of saline or drug on the challenge day, mice were anesthetized with Avertin (tribromoethanol) (0.25 mg/g, i.p.), and brain slices were prepared for whole-cell patch-clamp recordings. Slice preparation and recordings were made by an experimenter blinded to the treatment conditions. Mice (2–6 months old) were anesthetized with Avertin (tribromoethanol, 0.25 mg/g, i.p.), and the brains were rapidly removed and immersed in an ice-cold (4°C) slicing solution containing (in mM): 105 mM N-methyl-D-glucamine (NMDG), 105 mM HCl, 2.5 mM KCl, 1.2 mM NaH_2_PO_4_, 26 mM NaHCO_3_, 25 mM glucose, 2 mM thiourea, 1 mM Na-ascorbate, 3 mM Na-pyruvate, 0.5 mM CaCl_2_·4H_2_O, and 10 mM MgSO_4_·7H_2_O. The pH of the solution was titrated to 7.4 with concentrated HCl equilibrated with 95% O_2_ and 5% CO_2_, pH 7.4. Coronal corticostriatal slices (270 μm) were cut with a Leica VT1200 microtome. The slices were incubated, initially in the above slicing solution, for 10 min at 32°C, then for 40 min and subsequently at room temperature in artificial cerebrospinal fluid (ACSF) containing (in mM): 124 mM NaCl, 2.5 mM KCl, 1.2 mM NaH_2_PO_4_, 26 mM NaHCO_3_, 10 mM glucose, 2 mM CaCl_2_·4H_2_O, 1 mM MgSO_4_·7H_2_O, equilibrated with 95% O_2_ and 5% CO_2_, pH 7.4. The osmolality of all solutions was measured at 300–310 mOsm. Brain slices were transferred from the incubation chamber to the recording chamber and superfused with warmed (32°C), equilibrated (95% O_2_ and 5% CO_2_) ACSF at a flow rate of 2 ml/min. Whole-cell recordings were obtained from visually identified dorsolateral striatal neurons with an Olympus BX-51WI microscope (Olympus, Japan) equipped with Nomarski optics, infrared differential interference contrast (DIC) optics (900 nm) and epifluorescence. Whole-cell patch-clamp recordings were obtained from visually identified neurons using borosilicate glass pipettes (King Precision Glass, Inc., Glass type 8250) pulled (P-1000, Sutter Instruments) to a resistance of 2–5 MΩ when filled with the internal solution containing 131 mM K-Gluconate, 17.5 mM KCl, 9 mM NaCl, 10 mM HEPES, 1 mM EGTA, 1 mM Mg-ATP, 0.2 mM Na_2_-GTP, and 1 mM MgCl_2_. The pH was adjusted to 7.3 with KOH (290–300 mOsm). In all recording conditions, access resistance was monitored and cells were included for analysis only if the series resistance was <25 MΩ and the change of resistance was <15% over the course of the experiment. A Digidata 1440A digitizer and pClamp10 (Molecular Devices) were used for data acquisition and analysis, and signals were amplified with a Multiclamp 700B amplifier (Molecular Devices). A holding command of −70 mV was applied to the patched cell, and once the whole-cell mode was established, the cell was allowed to stabilize for 5–10 min. Both SPNs and ChIs were identified by cell-body size and shape and by intrinsic membrane properties. In current-clamp mode, light application evoked depolarization and action potentials in identified mCherry-positive ChIs in all except for one drug-treated, CalDAG-GEFI-GFP BAC;ChAT-Cre knock-in mouse that was therefore dropped from the study. In the single transgenic, virally injected ChAT-Cre knock-in mice and double transgenic CalDAG-GEFI-GFP BAC;ChAT-Cre knock-in mice, a blue LED pulse (10 ms) was used to stimulate ChIs. In the double BAC transgenic CalDAG-GEFI-GFP;ChAT-ChR2-EYFP mice, a hand-controlled shutter was briefly opened to activate the ChIs. For analysis of action potentials in SPNs, each SPN was maintained at resting membrane potential and injected for 800 ms with depolarization currents of 20–200 pA (enough current injection for adequate action potential firing). After a rest of 30 s, the same current sweep was repeated, this time with blue light application at 500 ms after the beginning of current injection. After 1 min of rest, another such pair of no-light/light current sweeps was given but with a slightly higher current injection to induce higher frequency firing. A total of 3 or 4 pairs of no-light/light current sweeps were given to each cell.

In recordings from two CalDAG-GEFI-GFP BAC;ChAT-Cre knock-in mice, and additional experiments with double BAC transgenic CalDAG-GEFI-GFP;ChAT-ChR2-EYFP mice, DHβE (1 mM; Tocris, Bristol, UK) was added to the perfusate after the initial recordings and slices were allowed to equilibrate for 30 min before repetition of action potential measurements.

### Electrophysiological data analyses

Data were obtained and recorded in ClampFit (Molecular Devices), and the output abf files were converted in MiniAnalysis (SynaptoSoft) to assign the timestamp of each spike. For spike density functions, a 1 kHz sampling rate was used. The value 1 was assigned at each sample that corresponded to a spike time, and the remaining samples were assigned the value 0. The resulting time series was then smoothed with a Hanning window whose half-width was 80 ms.

For the Grubbs' test to identify outlier interspike intervals (ISIs), trials of two sweeps (one without blue light pulse, one with) were selected based on the following criteria: (1) the combined pre-light baseline period contained at least 7 ISIs, (2) the pre-light period contained at least two ISIs (no trial failed this test after passing the first test), (3) the combined pre-light baseline ISIs were normally distributed according to the Lilliefors test with alpha = 0.001 (this low alpha value was chosen because of the small sample sizes under examination), and (4) the combined pre-light baseline ISIs did not contain any outliers as determined by the Grubbs' test. All but one neuron had at least one analyzable trial, although in most cases the first trials had too few spikes to analyze. The first ISI was discarded because in some instances it appeared to be longer than the remaining ones. To test for a significant “advanced spike” response, the minimum ISI recorded during the post-light period was added to the pre-light baseline set of ISIs, and the Grubbs' test for an outlier was performed. To test for a “pause” response, the Grubbs' test was performed again but with the maximum post-light ISI added to the pre-light baseline set. If the duration between the last spike of the trial and the termination of current injection was longer than the longest recorded ISI, it was used in place of the maximum ISI. As two Grubbs' tests were performed on each of 123 trials that met our criteria, we used the Bonferroni correction for 246 comparisons to set alpha = 0.0002, corresponding to a *p* level of 0.05 for the entire set of comparisons.

The Lilliefors statistical test was performed using the Matlab function “lillietest.” Other tests were performed by in-house code written around the Matlab functions “chi2cdf” (Chi squared tests) and “tinv” (Grubbs' test).

## Results

### Mice engaged in amphetamine-induced repetitive behaviors fail to respond to a sensory cue and exhibit striosome-enriched c-Fos induction

**Figure 1:**
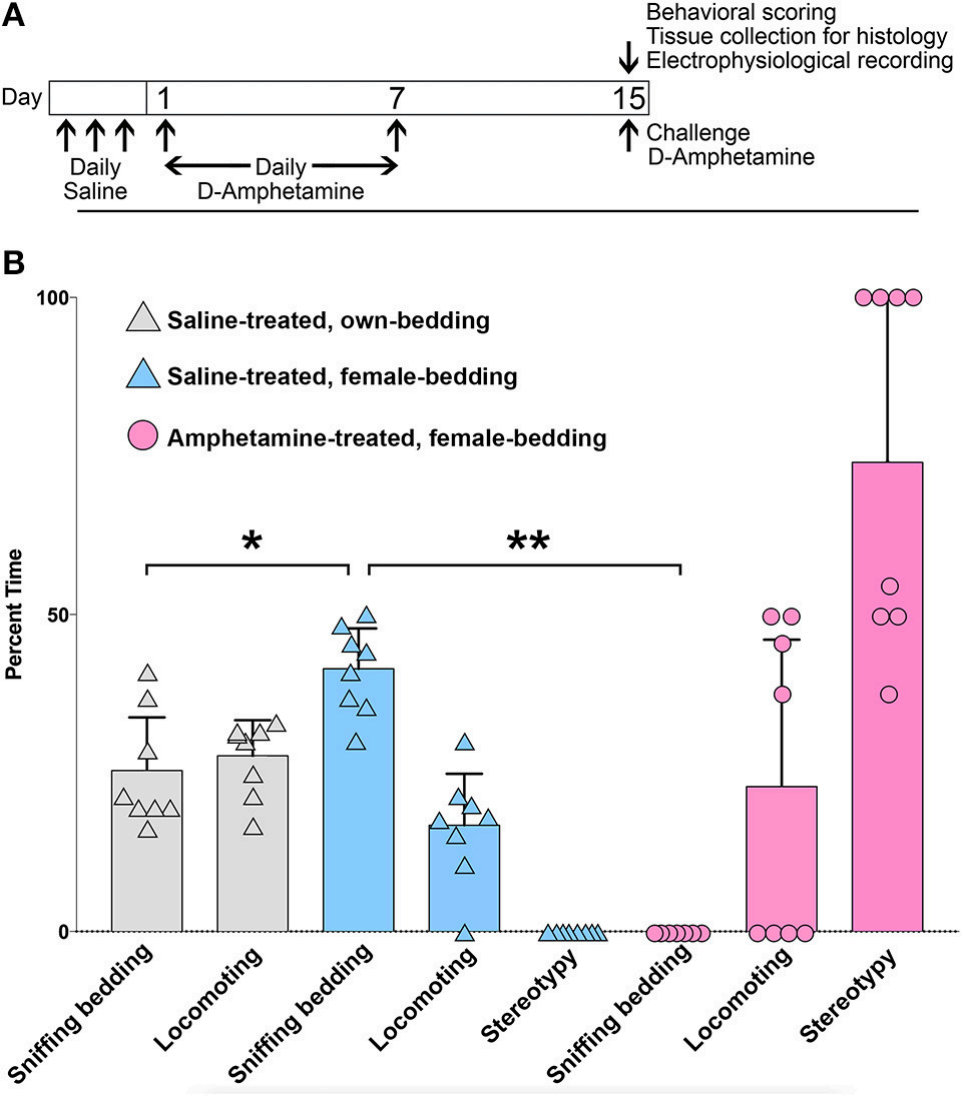
**Mice with amphetamine-induced stereotypy fail to engage with a salient sensory cue**. **(A)** Schedule of D-amphetamine or saline injections. **(B)** Analysis of behavior in saline-treated (*n* = 8) or D-amphetamine-treated mice (*n* = 8), for a 2 min videotaped interval at 30 min after injection, and 5 min after the introduction of bedding from female cages, or from their own home cage (for saline-treated only). The saline-treated mice interacted more with the female cage bedding than with bedding that was re-introduced from their own home cage (^*^p = 1.2 × 10^−3^, paired *t*-test). D-amphetamine-treated mice did not spend any time sniffing the female bedding (^**^p = 8.1 × 10^−11^, unpaired *t*-test, relative to saline-treated mice with female bedding). Error bars show standard deviations.

To test whether mice with drug-induced stereotypies fail to respond to salient sensory cues, we measured the response of single-housed, amphetamine-treated male mice to the introduction of bedding from female cages to their own cage. To induce stereotypy, mice were treated for 7 days with high-dose D-amphetamine (7.0 mg/kg/day, i.p.), followed by 7 days of no-treatment and then a challenge dose (7.0 mg/kg, i.p.), as previously described (Figure 1A; Crittenden et al., 2014). Control mice were treated in parallel with vehicle (saline) injections only. On the challenge day, at 30 min after injection, we introduced a ball of used bedding taken from multiple cages of females. We scored the times the mice spent sniffing the bedding, locomoting or engaging in confined stereotypic behaviors based on a video taken 5 min after the introduction of the female bedding. Control mice spent between 30 and 50% of the time sniffing the female bedding (Figure 1B). We compared this level to the times that they interacted with bedding that had been taken from their own home cage and found that they spent significantly more time interacting with the female cage bedding (Figure 1B), supporting the interpretation that female cage bedding is a salient cue. By contrast, mice that were treated with repeated amphetamine spent most of their time engaging in stereotypic behaviors, sometimes interrupted by running bouts, and did not interact with the female cage bedding at all (Figure 1B).

**Figure 2:**
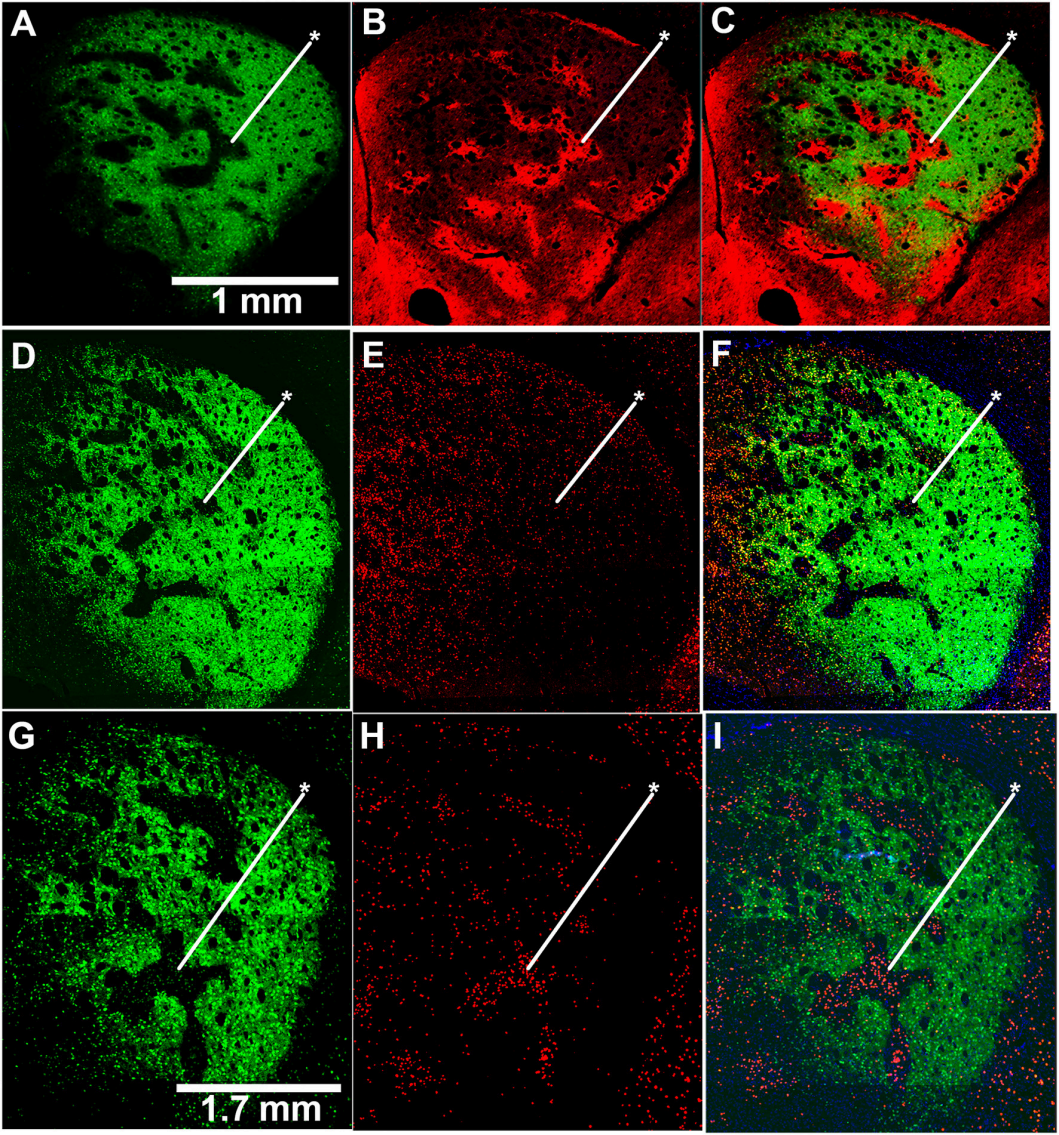
**Induction of c-Fos expression is striosome-predominant in D-amphetamine-treated mice**. **(A–C)** Image of GFP fluorescence in a coronal hemisection through the left striatum of a CalDAG-GEFI-GFP BAC transgenic mouse used to differentiate striosomes and matrix **(A)** and co-immunolabeling for the striosome marker, MOR1 **(B)**, with merged image **(C)**. **(D–I)** Sections from transgenic mice treated with repeated saline **(D–F)** or repeated D-amphetamine **(G–I)** showing CalDAG-GEFI-GFP fluorescence **(D,G)** and c-Fos co-immunolabeling (red nuclei) **(E,H)**. Merged images **(F,I)** illustrate the striosome-predominant c-Fos induction by the D-amphetamine challenge injection. Asterisks indicate examples of striosomes. Similar results were observed in 4 saline-treated and 4 D-amphetamine-treated mice.

To test whether the mice exhibiting drug-induced stereotypy had imbalanced striosome-to-matrix ratios of immediate-early gene activation, as occurs in rats and monkeys (Canales and Graybiel, 2000; Saka et al., 2004; Horner et al., 2012; Jedynak et al., 2012), we imaged c-Fos expression in striatal sections taken from D-amphetamine-treated mice (*n* = 4) compared to saline-treated mice (*n* = 4). To detect the striosome and matrix compartments, we used BAC transgenic mice (Gong et al., 2007) with an enhanced GFP reporter for the matrix-enriched signaling molecule, CalDAG-GEFI (Kawasaki et al., 1998; Crittenden et al., 2016). The compartmental selectivity of CalDAG-GEFI-GFP expression followed gradients across the striatum, with the compartmental borders being easiest to identify in the central and lateral parts of the caudoputamen (Figures 2A–C). CalDAG-GEFI-GFP mice were treated with D-amphetamine or saline, according to the schedule shown in Figure 1A, and brain tissue was taken for analysis 90 min after the challenge injection. Mice that received amphetamine injections, but none of the saline-treated mice, were engaged in stereotypic behaviors at the time of euthanasia. Saline-treated control mice showed low levels of c-Fos expression in cell nuclei distributed across both compartments in the striatum (Figures 2D–F). By contrast, the striatum of the D-amphetamine-treated mice showed strong expression of c-Fos in the striosomes, with a lower density of c-Fos-positive nuclei in the surrounding matrix compartment (Figures 2G–I).

### Cholinergic interneurons of the striatum project their axons into both striosome and matrix compartments of both control and drug-treated mice

**Figure 3:**
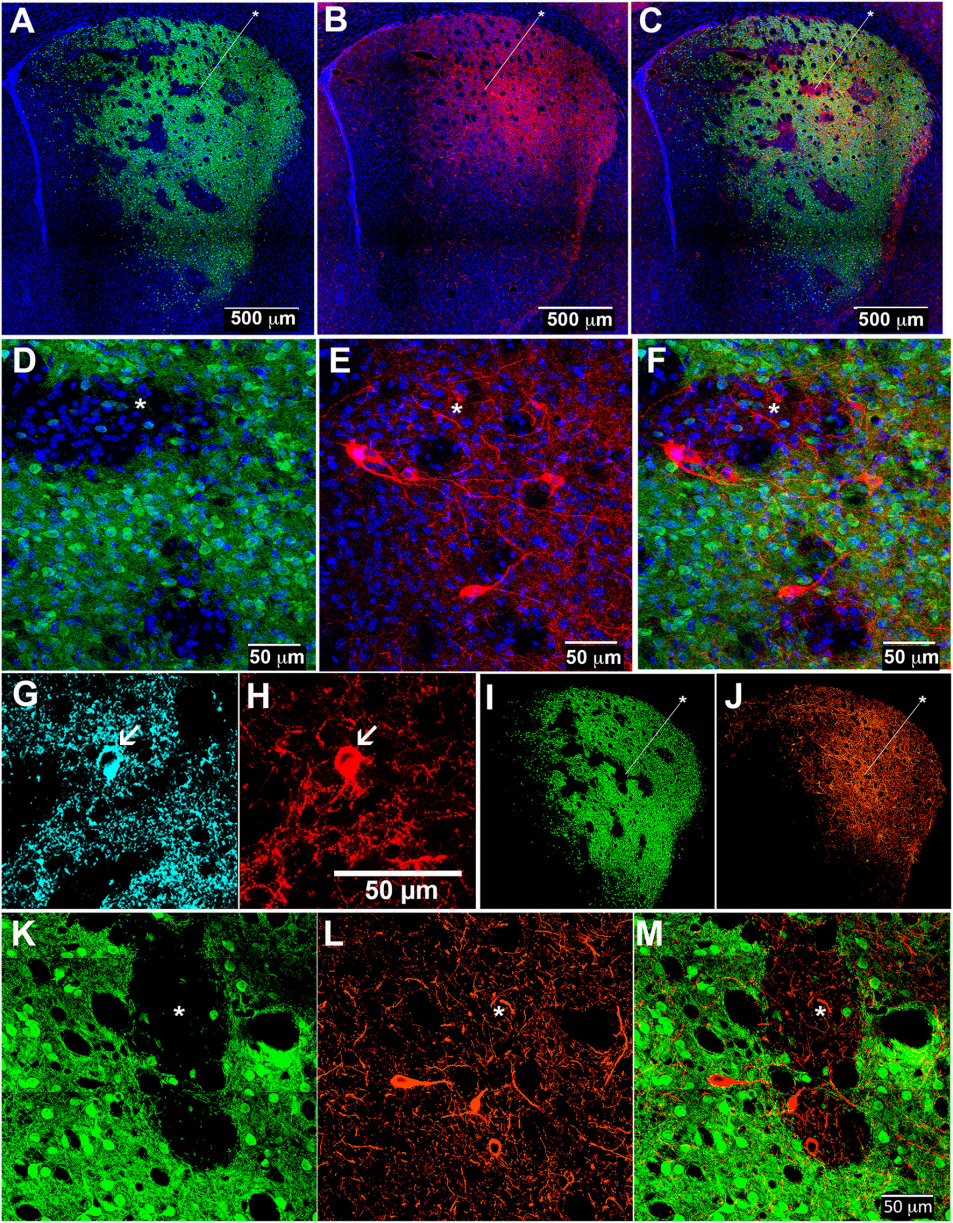
**Cholinergic interneuron processes in striosomes and matrix of control and D-amphetamine-treated mice**. Striatal sections from CalDAG-GEFI-GFP BAC;ChAT-Cre knock-in mice labeled for GFP in the matrix **(A,D,I,K)**, AAV5 Cre-dependent ChR2-mCherry in ChIs **(B,E,J,L)**, and merged images from double transgenic mice **(C,F,M)** treated with saline **(A–F)** or repeated D-amphetamine **(I–M)**. Immunofluorescence (cyan) for VAChT **(G)** confirms the cholinergic identity of an AAV-infected mCherry-positive neuron **(H)**. Asterisks indicate examples of striosomes.

To allow simultaneous detection of striatal ChIs and striosome-matrix compartments, we crossed the CalDAG-GEFI-GFP BAC mice to ChAT-Cre knock-in mice (Table 1). In the ChAT-Cre knock-in mice, Cre recombinase is fused to the end of the choline acetyltransferase gene at its endogenous locus (Rossi et al., 2011) so as not to evoke over-expression of the nearby VAChT gene, as has been reported to occur in ChAT BAC transgenic mice (Kolisnyk et al., 2013; Crittenden et al., 2014). We labeled ChIs in the double transgenic mice by intrastriatal injections of an AAV5 virus encoding a Cre-dependent transgene for the mCherry fluorophore fused to channelrhodopsin [Ef1αDIOhChR2(H134R)-mCherry] (Mattis et al., 2012). The infected mCherry-positive cells (Figures 3A–F) resembled ChIs in that they were large and appeared scattered across the striatum, contained substantial extra-nuclear cytoplasm in the soma, and had multiple spots in the nucleus that stained for the chromatin-marker DAPI (Matamales et al., 2009). We confirmed that these mCherry-positive neurons expressed the endogenous cholinergic cell marker VAChT in their cell bodies (in cyan in Figures 3G,H). Very fine ChI fibers, presumably axons, were present in both the striosomes and matrix (Figure 3F). Thicker, presumably dendritic, processes were concentrated in the matrix, with some running along compartment borders and occasionally crossing the borders (Figure 3F).

To be sure that the ChI innervation of both striosomes and matrix was maintained after repeated D-amphetamine treatment, we injected the double transgenic mice intrastriatally with the AAV5-ChR2-mCherry, waited for 2 weeks, and then treated the mice with D-amphetamine according to the standard schedule (Figure 1A) prior to collection of brain tissue on the challenge day. As in control mice, cholinergic neurons were evident along with dense neuropil from infected ChIs (Figures 3I–M).

**Figure 4:**
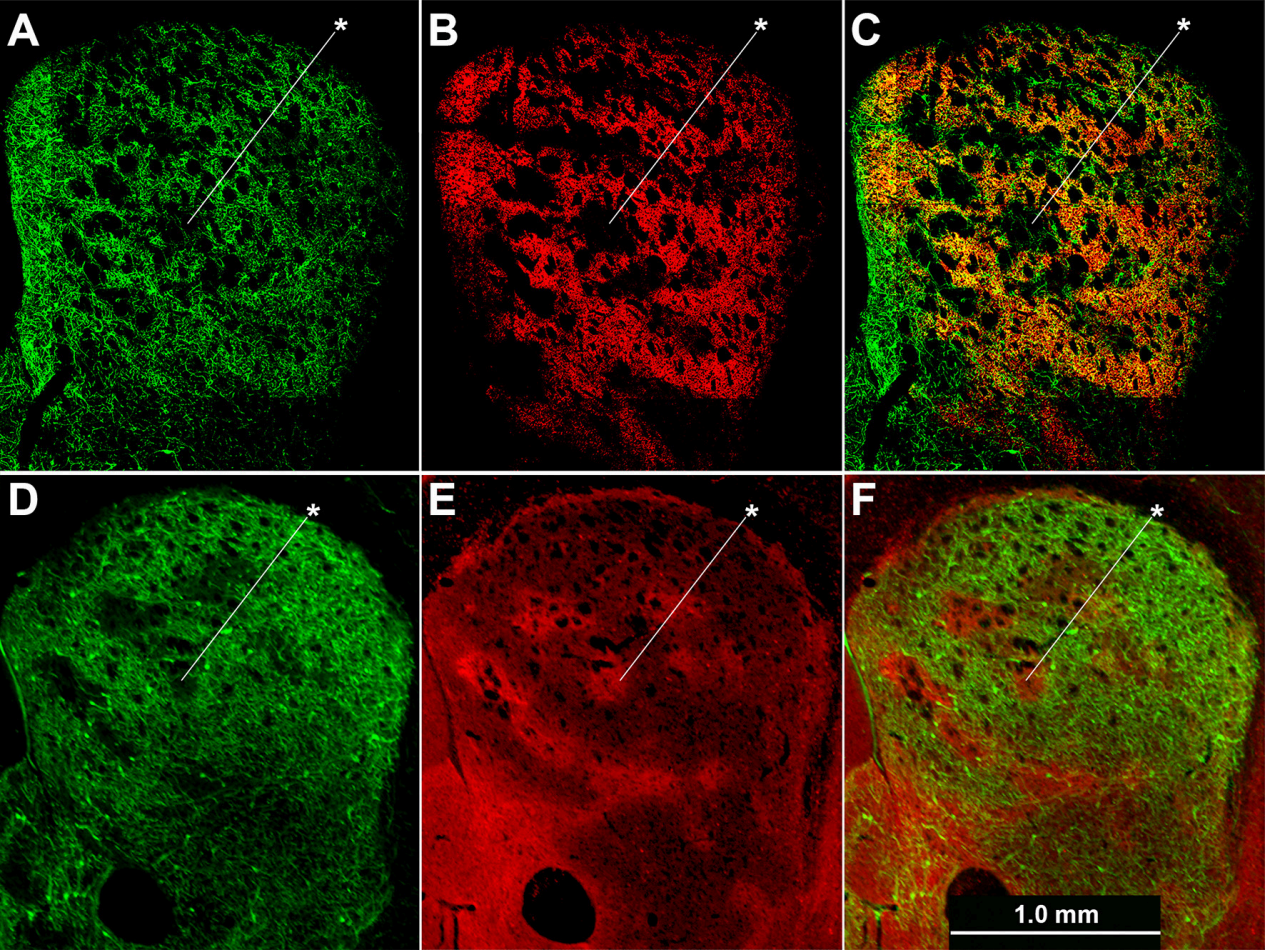
**Cholinergic neuropil is not homogenously distributed across the striatum**. Hemi-sections through the left striatum from ChAT-Cre;Ai32-EYFP knock-in **(A–C)** and ChAT-ChR2-EYFP BAC **(D–F)** transgenic mice showing fluorescence labeling of global cholinergic neuropil in green **(A,D)** relative to co-immunolabeling in red for the matrix marker CalDAG-GEFI **(B)** or striosome marker MOR1 **(E)**. **(C,F)** Merged images. Asterisks indicate examples of striosomes.

Cholinergic neuropil, though present in both compartments, was not homogenously distributed across the striatum, in accord with previous findings (Graybiel et al., 1986). The cholinergic neuropil distribution was most clearly evident in mice with global labeling for cholinergic cell reporters (double transgenic knock-in ChAT-Cre;Ai32-EYFP mice and single BAC transgenic ChAT-ChR2-EYFP mice, (Table 1; Figure 4). Striosomes, especially in the medial striatum, were often visible as darker regions with reduced densities of large cholinergic processes, both in the ChAT-Cre mice with intrastriatal viral injections and in the ChAT-Cre;Ai32-EYFP knock-in and ChAT-ChR2-EYFP BAC mice.

### Cholinergic interneuron activation induces a pause or a spike advance to disrupt SPN firing patterns

**Figure 5:**
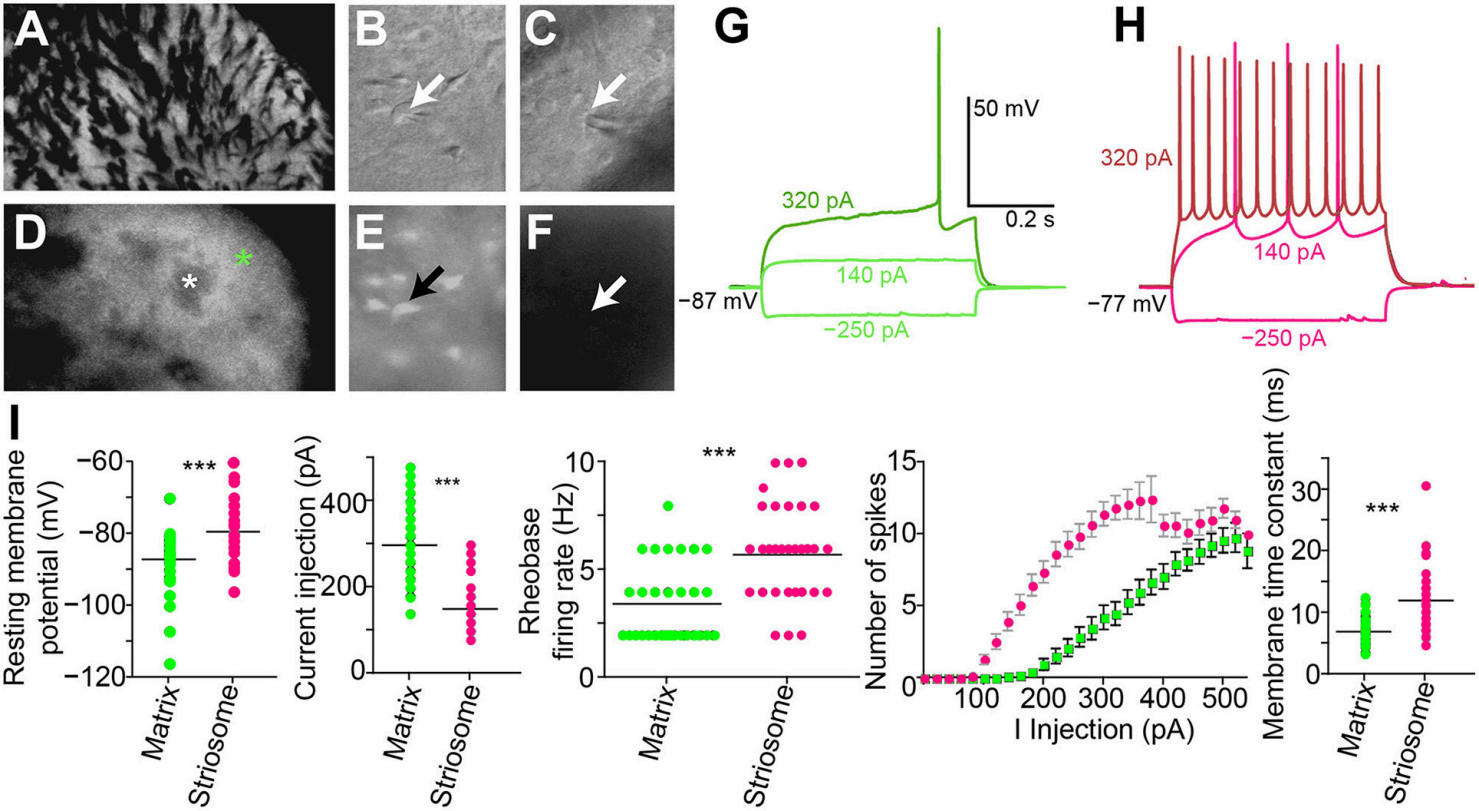
**Increased excitability in striosomal SPNs**. **(A–F)** Photomicrograph of the left dorsolateral striatum from a CalDAG-GEFI-GFP BAC transgenic mouse with transmitted light (differential interference contrast) **(A–C)** and epifluorescence **(D–F)** microscopy. Green asterisk denotes matrix, and white denotes a striosome in **(D)**. Patch-clamp recording pipette in contact with a GFP-positive matrix cell **(B,E)** or a striosomal cell **(C,F)**. Black and white arrows point to cell somata patched with a recording pipette. **(G,H)** Current-voltage curve traces from a matrix SPN with rheobase (320 pA) in dark green **(G)** and a striosomal SPN with rheobase (140 pA) in magenta **(H)**. **(I)** Striosomal SPNs (magenta) are more excitable than matrix SPNs (green) as measured by resting membrane potential, current injection for rheobase, spike-firing frequency at rheobase, IV curve, and membrane time constant. ^***^p < 0.0005 by Mann-Whitney Rank Sum test.

We made whole-cell patch-clamp recordings from GFP-positive striatal neurons in the matrix and from GFP-negative striatal neurons within striosomes of CalDAG-GEFI-GFP BAC mice (Figures 5A–F). With the goal of testing for cholinergic modulation of excitatory inputs to SPNs, we measured the amount of current injection required to induce repetitive spiking activity (rheobase) in striosomal and matrix SPNs. SPNs typically fired regularly at current injection levels above rheobase, and striosomal neurons were more excitable than matrix neurons in the striatal slices taken from mature mice (*n* = 33 striosomal SPNs, *n* = 32 matrix SPNs, from 16 mice; Figures 5G–I), as previously reported for slices taken from young mice and rats (Kawaguchi et al., 1989; Miura et al., 2007; Smith et al., 2016).

**Figure 6:**
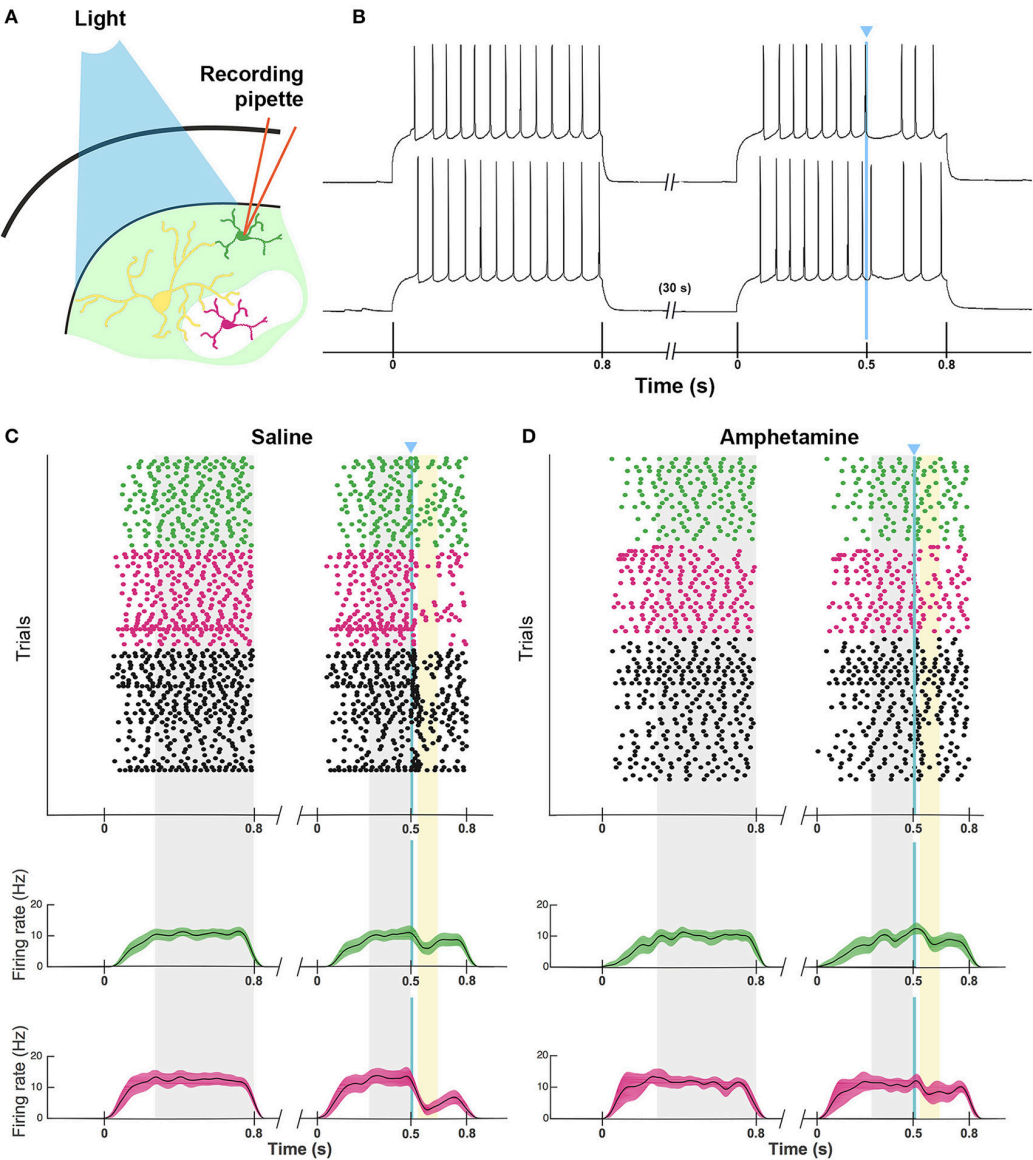
**ChI activation disrupts spiking pattern in striosomal and matrix SPNs, but not after D-amphetamine treatment. (A)** Cartoon of slice recording set-up with ChI (yellow), matrix SPN (green) and striosomal SPN (magenta) in a mouse with CalDAG-GEFI-GFP labeling in the matrix and blue light responsive ChR2 in the ChIs. **(B)** A timeline showing the schedule of paired 800 ms current injections used, with light pulse during the second sweep. Examples of a pause (top trace) and a spike advance followed by a pause (bottom trace) after optogenetic activation of ChIs (at blue line). **(C,D)** Raster plots (upper) and spike density functions (lower) of all of the spikes recorded across all sweeps (one trial per row) from saline-treated **(C)** and D-amphetamine-treated **(D)** mice. In the raster plots, matrix SPN spikes are in green, striosomal SPN spikes in magenta and compartmentally unidentified SPN spikes from single transgenic ChAT-Cre knock-in mice in black. Spike density functions are averaged over all trials from identified matrix (green) and striosomal (magenta) SPNs. The blue line indicates the onset of 10 ms light stimulus. Gray shading denotes the “baseline” period (270–800 ms after the start of the first current injection) and the “pre-light” period (from 270 ms after the start of the second current injection until light onset). Yellow shading denotes the “response” period (from 40 to 150 ms after light onset).

**Figure 7:**
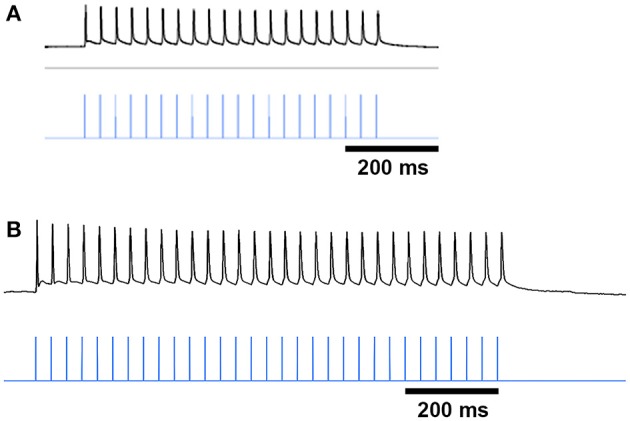
**ChI spike responses to blue-light stimulation**. Black traces show that ChIs, expressing ChR2-mCherry from AAV5 injections, fired single action potentials in response to 10 ms blue light pulses (vertical blue lines) in striatal slices from saline-treated **(A)** and D-amphetamine-treated **(B)** CalDAG-GEFI-GFP BAC;ChAT-Cre knock-in mice.

As a direct test of whether the striatal ChIs can modulate activity in striosomal and matrix SPNs, we then made whole-cell patch-clamp recordings in coronal slices through the striatum of double transgenic CalDAG-GEFI-GFP BAC;ChAT-Cre knock-in mice in which intrastriatal injections of AAV5-ChR2-mCherry had been made (Figures 3A–F, 6A). At 2 weeks after intrastriatal virus injection, the double transgenic CalDAG-GEFI-GFP BAC;ChAT-Cre knock-in mice began daily saline injections in order that they could serve as controls for comparison to sibling mice treated with D-amphetamine (Figure 1A). After the final injection, we prepared brain slices and applied wide-field, 10 ms blue light pulses to the striatal slices (Figure 6A). This treatment reliably generated an action potential in ChIs of both saline-treated and amphetamine-treated mice (Figure 7A). To test how ChI activation modulated SPN activity, we induced epochs of regular SPN spiking activity by 800 ms current injection sweeps. We then compared SPN responses to current injection alone to the responses elicited 30 s later by the same current injection combined with a 10 ms light pulse beginning at 500 ms after the start of current injection (Figure 6B). Most neurons in the control group exhibited a pause or marked reduction in spiking following the light pulse, and some also showed a single spike at short latency after light application, typically followed by a pause (Figures 6B,C; *n* = 5 ChAT-Cre knock-in mice with viral expression of ChR2, 35 striatal neurons, 12 from striosomes, 10 from matrix, and 13 from mice with no matrix-GFP label). These effects were found in both single transgenic ChAT-Cre knock-in mice and double transgenic CalDAG-GEFI-GFP BAC;ChAT-Cre knock-in mice (Figure 6C). To insure that the responses were not artifacts of light application, we measured the SPN responses in single transgenic CalDAG-GEFI-GFP BAC mice with no ChR2 expression. In these control mice, the light application did not disrupt firing at all (data not shown).

**Figure 8:**
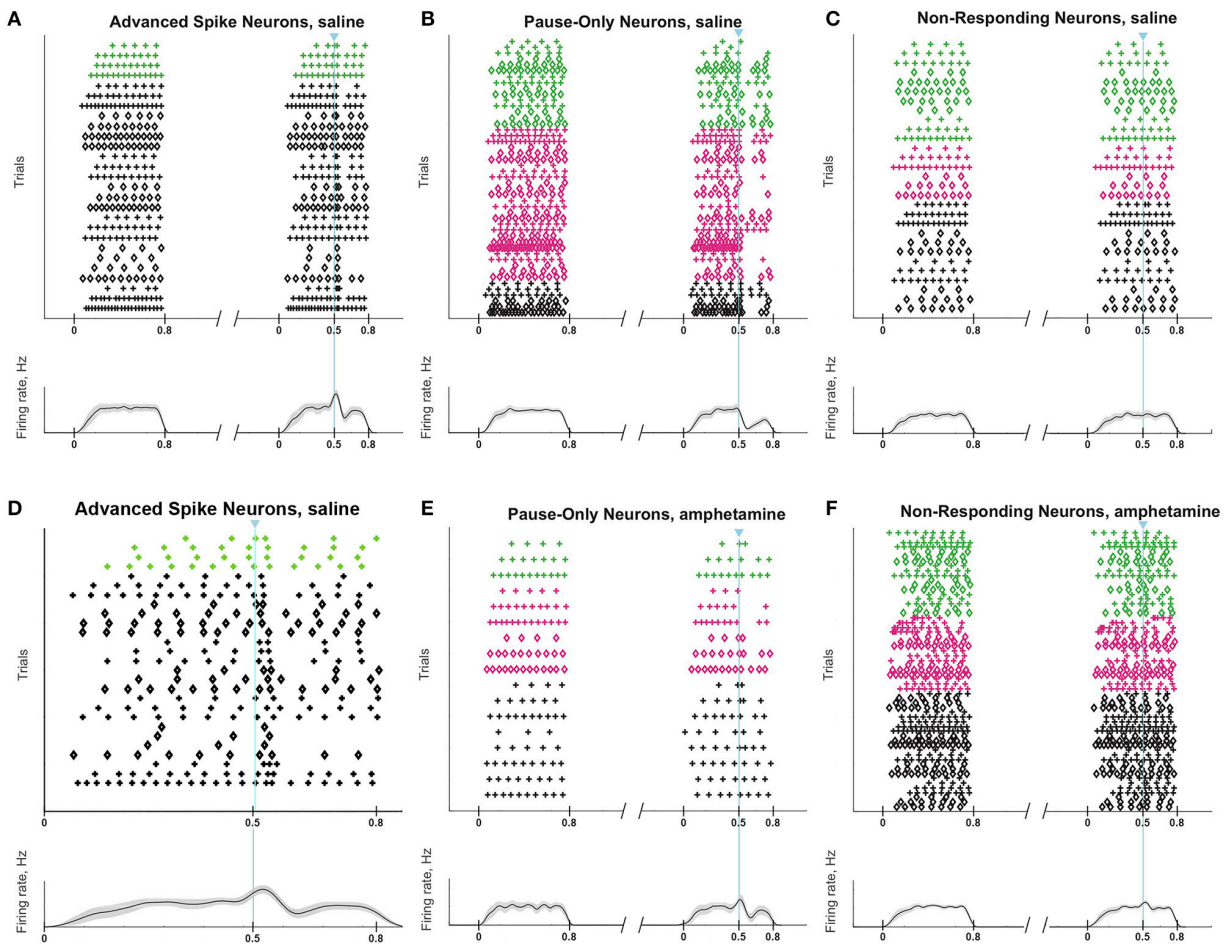
**ChI activation induces a pause or a spike that disrupts SPN spike-timing patterns**. Raster plots (top) and trial-averaged spike density functions (bottom) grouped according to neuronal response type. SPN types are color coded as in Figure 6. To show which trials belong to an individual SPN, spikes from odd-numbered neurons within each color group are plotted as plus signs, and those from even-numbered as diamonds (one trial per row). **(A–C)** SPNs recorded in saline-treated mice, showing advanced spike response **(A)**, pause response **(B)** or no response **(C)**. **(D)** Expanded time scale showing SPNs with advanced spike response in saline-treated mice. **(E,F)** SPNs recorded in D-amphetamine-treated mice, showing pause response in 4 neurons **(E)** or no response **(F)**.

Responses of a given SPN to the ChI activation were generally consistent across different trials at different current intensities, as shown in Figures 8A–C. SPNs typically exhibited one of three response types: advanced spike followed by a pause, pause only (without advanced spike), and non-responsive. The pause response was observed more frequently than the advanced spike response, but there was one exceptional neuron that responded to the light with advanced spikes but no pause (Table 2). In neurons with advanced spikes, the extra spikes were limited to a time window of about 40 or 50 ms after light onset (Figure 8D). To quantify these differences, we developed a statistical analysis to detect both increases and decreases in firing rate on a trial-by-trial basis. For each trial, ISIs were calculated for spikes in three time windows (Figure 6C): the first current injection (“baseline”), the portion of the second current injection preceding the light (“pre-light”), and the remainder of the second current injection (“post-light”). We then used Grubbs' test (see Materials and Methods) for outliers to determine whether the shortest and/or the longest post-light ISIs could be considered outliers in the context of the pre-light baseline ISIs. By this method, we found that both the pause and the advanced spike responses (Table 2) were statistically significant.

**Table 2:** **Response types of neurons in saline-treated control mice**.

**35 analyzed neurons**	**Matrix (*n* = 10)**	**Striosomal (*n* = 12)**	**Unidentified (*n* = 13)**
Pause	7	10	8
Advanced	1	0	7
Both	1	0	6
Neither	3	2	4

*If a neuron showed a statistically significant pause response in at least one trial, then it was counted as having a pause response, and similarly for advanced spike responses*.

### Striosomal SPNs can be more responsive to ChI modulation than matrix SPNs

To calculate the statistical significance of the striosomal and matrix SPN responses to light, we compared the total numbers of spikes in the response period from 40 to 150 ms after light onset to the numbers of spikes in the baseline and pre-light time windows (Figure 6C). We added the spike counts in the baseline and pre-light windows, and then performed a chi-squared test with Yates' correction comparing the actual spike counts in the response window and combined pre-light baseline window with the values expected under the null hypothesis that the light had no effect. The entire procedure was repeated separately for identified striosomal SPNs and identified matrix SPNs (Table 3). Striosomal SPNs as a group showed a significant effect of the light at a substantially lower *p* level than did the matrix group (*p* = 1.2 × 10^−7^ vs. *p* = 0.009). To verify that this difference reflected a stronger response to the light in striosomal SPNs, we performed a chi-squared test for dependence between the effects of neuron type (striosomal vs. matrix) and time window (pre-light baseline vs. response). The null hypothesis of no difference between striosomal and matrix SPNs was rejected with a significance level of *p* = 0.016 for the slice recordings from saline-treated mice. When aggregating spike counts across different neurons, the results will be dominated by the highest firing neurons. There were 3 out of 22 compartment-identified SPNs in the saline-treated group and 1 out of 14 compartment-identified SPNs in the amphetamine-treated group that had spike counts during the combined pre-light baseline window that were more than 1.5 times the aggregated median value. We repeated the statistical analysis after excluding these neurons (Table 4) and found with a chi-squared test that the group of striosomal SPNs still showed a trend to be more significantly affected than the matrix SPNs, but now with a non-significant *p* = 0.14. Just as before the exclusion of the more highly firing neurons, both the striosomal and matrix SPNs were found to be responsive to ChI-mediated modulation (*p* = 9 × 10^−5^ for striosomal vs. *p* = 0.024 for matrix SPNs).

**Table 3:** **Chi-squared test results for effect of light on total spike count**.

***p***	**Striosomal**	**Matrix**
Saline	1.2 × 10^−7^	0.009
Drug	0.30	0.32

**Table 4:** **Chi-squared test results for effect of light after excluding high-firing neurons**.

***p***	**Striosomal**	**Matrix**
Saline	9 × 10^−5^	0.024
Drug	0.30	0.44

### Firing activity of striosomal and matrix SPNs is resistant to ChI-mediated disruption in mice with drug-induced stereotypy

To test whether SPNs from mice in a state of severe repetitive behavior exhibit abnormalities in the ChI-to-SPN signaling microcircuit, we recorded in slices taken from mice engaged in amphetamine-induced stereotypy (*n* = 4 ChAT-Cre knock-in mice with viral expression of ChR2, 24 striatal neurons, 7 from striosomes, 7 from matrix, and 10 from mice with no matrix-GFP label). On the amphetamine challenge day, we isolated brain tissue at the 30 min time-point after D-amphetamine injection and prepared striatal sections for whole-cell recording (Figure 1A). The drug-treated mice were confirmed to be in a state of stereotypy at the time of tissue collection. ChIs in the dorsal striatum of slices from the drug-treated mice responded reliably to light stimulation (Figure 7B) except in one mouse; data from this mouse were not included in the analyses. We did not analyze baseline activity of ChI activity in control or drug-treated mice but, rather, tested whether SPNs responded differently to the single spike induced by optogenetic stimulation of the ChIs.

The raster plots and spike density plots of the striosomal and matrix SPN responses to ChI stimulation showed a severely blunted SPN response in the slices from D-amphetamine-treated mice (Figure 6D). We evaluated the significance of the responses by performing chi-squared tests with Yates' correction to compare the spike counts in the post-light time-window and the baseline plus pre-light spike counts with the values expected under the null hypothesis that the light had no effect. These tests showed that neither striosomal nor matrix SPN firing frequencies showed a significant response to the light (*p* = 0.4 for striosomal SPNs and *p* = 0.4 for matrix SPNs). We performed a chi-squared test for dependence between the effects of neuron type (striosomal vs. matrix) and time window (pre-light baseline vs. response) and found that no difference was apparent (*p* = 0.9). Although there were some neurons that showed a response to the light after D-amphetamine treatment (Table 5; Figure 8E), the proportion of SPNs that responded was much lower in the drug-treated group (Figure 8F) than in the saline-treated group (22% vs. 74% respectively), and this difference was significant by Fisher's exact test (*p* = 1.3 × 10^−4^).

**Table 5:** **Response types of SPNs in D-amphetamine-treated mice**.

**23 analyzed neurons**	**Matrix (*n* = 7)**	**Striosomal (*n* = 7)**	**Unidentified (*n* = 9)**
Pause	1	2	2
Advanced	0	0	1
Both	0	0	1
Neither	6	5	7

**Figure 9:**
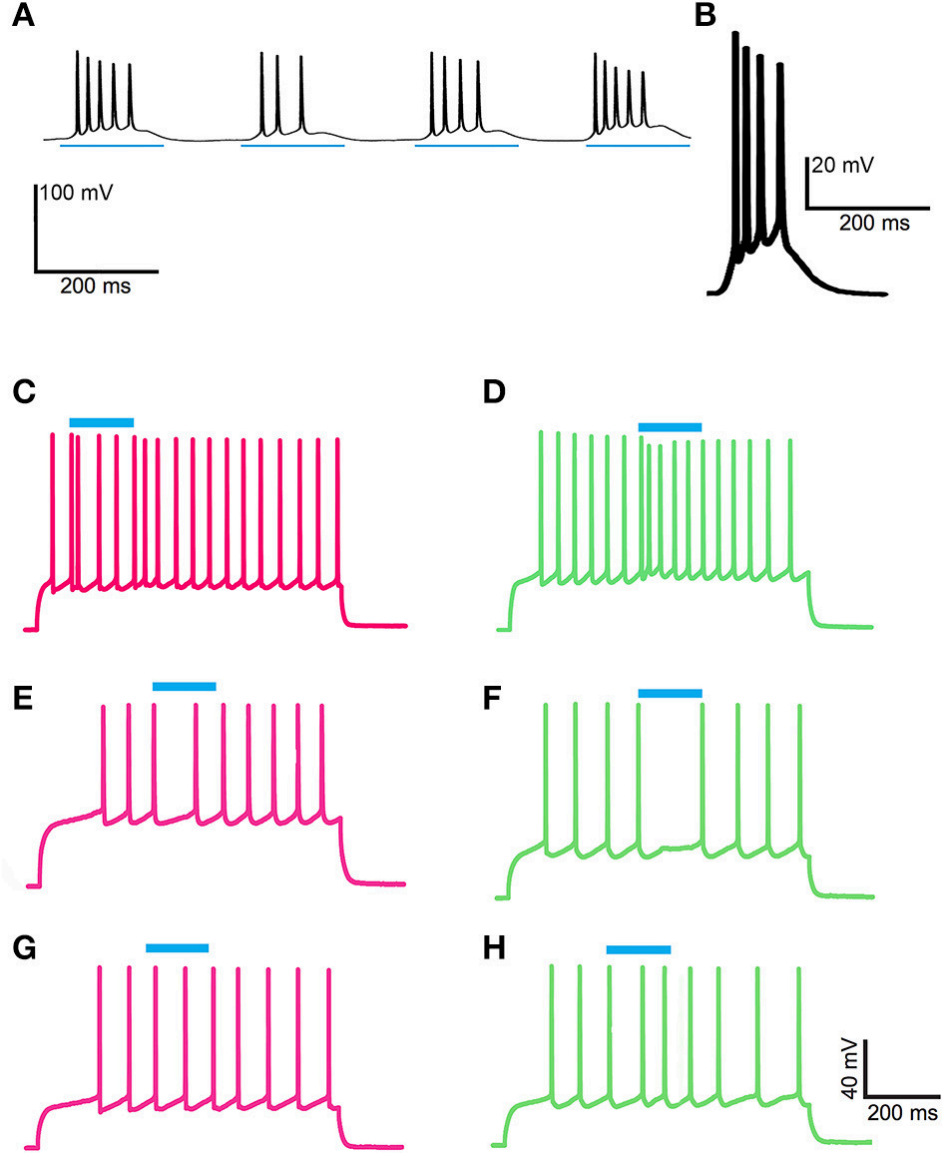
**ChI activation disrupts spiking patterns in striosomal and matrix SPNs by a pause or an advanced spike in ChAT-ChR2-EYFP BAC mice and the effect is blocked by D-amphetamine treatment. (A,B)** ChIs showed a burst of firing in response to shutter-controlled blue light application (designated by horizontal blue lines) in striatal sections from saline-treated **(A)** and D-amphetamine-treated **(B)** ChAT-ChR2-EYFP BAC mice. **(C–F)** Current-induced spiking in striosomal (magenta, **C,E**) and matrix (green, **D,F**) SPNs is disrupted by an advanced spike **(C,D)** or a pause **(E,F)** after blue-light application. **(G,H)** The striosomal (magenta, **G**) and matrix (green, **H**) SPN responses to ChI activation with blue light is blocked in sections from D-amphetamine-treated mice.

To confirm that our results were not unique to virally mediated expression of ChR2, we also made recordings in double BAC transgenic CalDAG-GEFI-GFP;ChAT-ChR2-EYFP mice that had global cholinergic expression of a ChR2 transgene (*n* = 5 mice) and that were treated with either repeated saline or repeated D-amphetamine (Figure 1A). For these tests, we administered blue light with a manually controlled shutter, thus inducing multiple spikes in the ChIs of saline-treated and D-amphetamine-treated mice (Figures 9A,B). We recorded from 7 SPNs that were CalDAG-GEFI-GFP-positive (matrix) and 7 SPNs that were in CalDAG-GEFI-GFP-negative zones (striosomes). In the saline-treated preparations, we observed that the spiking pattern of both striosomal and matrix SPNs could be disrupted by a pause or by a spike advance (Figures 9C–F), as we had found for the SPN responses in ChAT-Cre knock-in mice with virally expressed ChR2. However, in slices taken from the amphetamine-treated mice expressing severe D-amphetamine-induced stereotypy (*n* = 5 mice, 29 striatal SPNs, 13 from striosomes, 16 from matrix), the striosomal and matrix SPNs no longer responded to light (Figures 9G,H). Thus, ChI activation normally produced a pause or spike-advance in the regular firing pattern of striosomal and matrix SPNs in brain slices from two different lines of transgenic mice but these effects were blocked in slices taken from amphetamine-treated mice exhibiting drug-induced stereotypic behavior.

### D-amphetamine treatment did not induce a chronic, baseline failure in the ability of ChIs to disrupt SPN firing patterns

**Figure 10:**
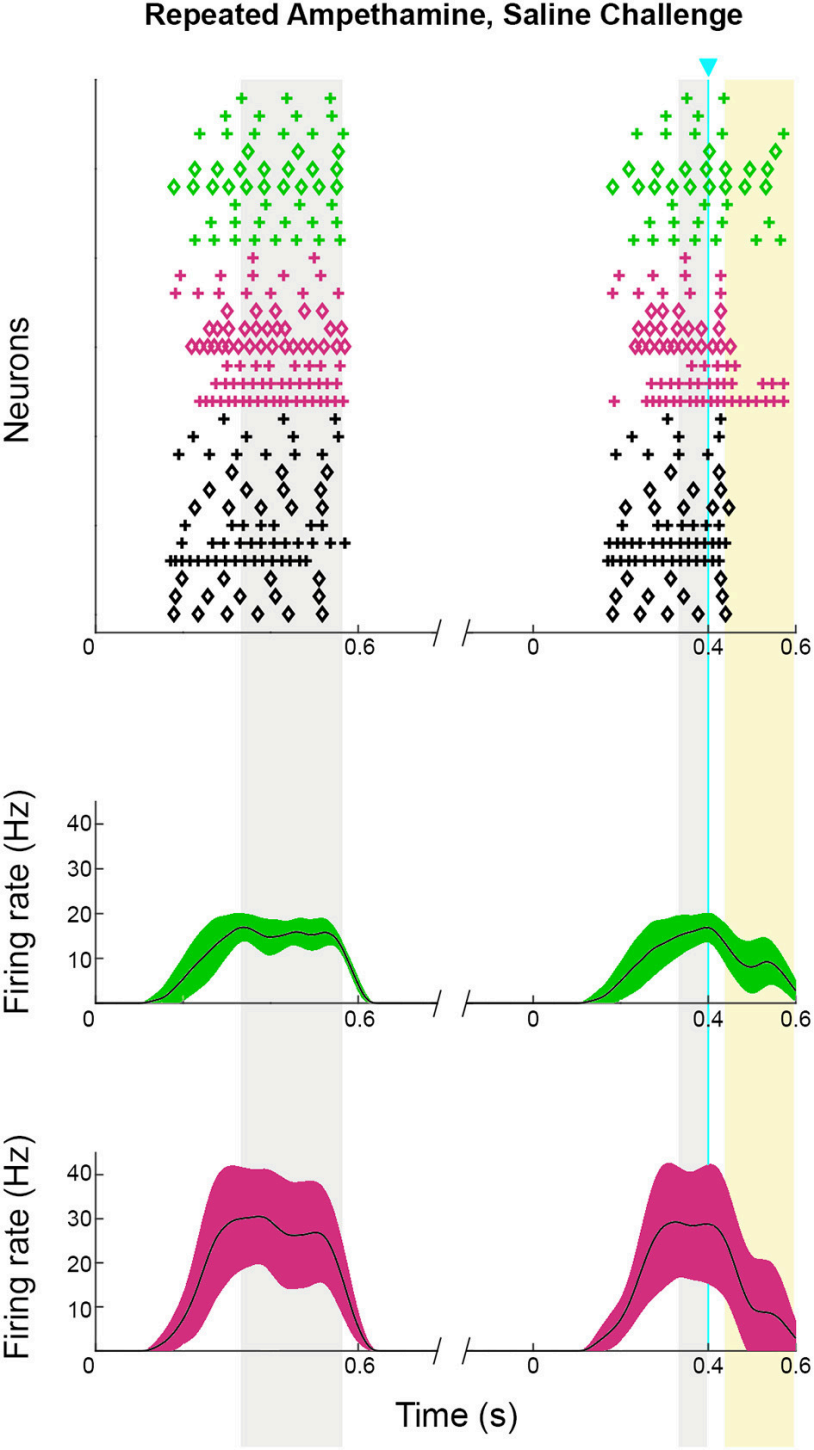
**Striosomal and matrix SPN responses to ChI activation were maintained in mice that received repeated D-amphetamine treatment but were challenged with saline only**. Raster plots (upper) and spike density functions (lower) of all of the spikes recorded across all sweeps (one trial per row). In the raster plots, matrix SPN spikes are in green, striosomal SPN spikes in magenta and compartmentally unidentified SPN spikes from single transgenic ChAT-Cre knock-in mice in black. To show which trials belong to an individual SPN, spikes from odd-numbered neurons are plotted as plus signs, and those from even-numbered as diamonds. Spike density functions are averaged over all trials from identified matrix (green) and striosomal (magenta) SPNs. The blue line indicates the onset of 10 ms light stimulus. Gray shading denotes the “baseline” and “pre-light” periods and yellow shading denotes the “response” period.

Repeated methamphetamine treatments can produce prolonged changes in baseline striatal acetylcholine signaling (Bamford et al., 2008). To test whether the ChI to SPN signaling phenotype that we observed in drug-treated mice was due to a baseline change in SPN responsivity, we treated mice with the same 7-day drug treatment schedule but challenged them with saline, not amphetamine, prior to recording (*n* = 2 mice, 10 striatal neurons, 3 from striosomes, 3 from matrix, and 4 from mice with no matrix-GFP label). As before, we harvested brain slices at 30 min after the challenge, and we confirmed, in every slice, that ChIs responded to the 10 ms light pulse with a spike. Optogenetic stimulation of ChIs in striatal slices taken from these mice appeared to disrupt SPN spike-timing just as in mice that had never been exposed to drug (Figure 10). A chi-squared test comparing spike density in the baseline and pre-light periods to the period after light application (periods highlighted in Figure 10) showed that there was a significant effect in each mouse that was tested (*p* = 0.0013 and *p* = 4.2 × 10^−5^), and when the results from both mice were combined, the value was *p* = 4.6 × 10^−7^.

### The disruption of striosomal and matrix SPN firing pattern by ChI activation is dependent on nicotinic acetylcholine receptors

**Figure 11:**
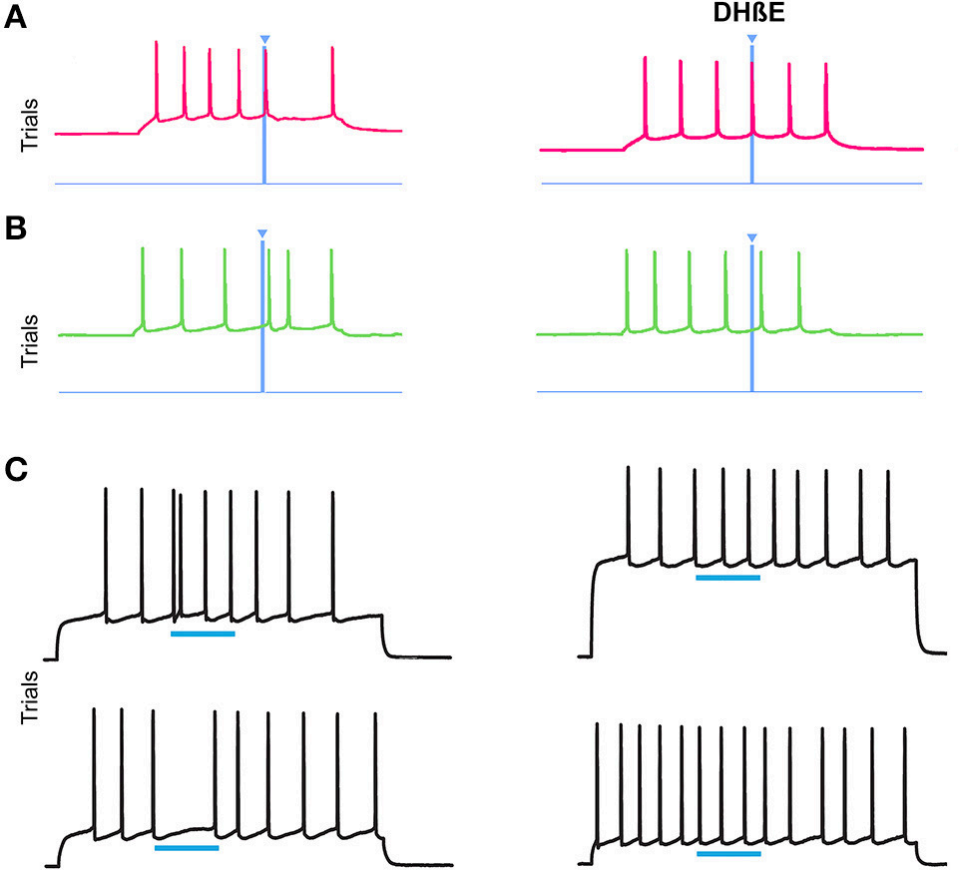
**Nicotinic acetylcholine antagonism blocks the ChI disruption of SPN spiking activity. (A,B)** Current-induced spiking in striosomal **(A)** and matrix **(B)** SPNs from CalDAG-GEFI-GFP BAC;ChAT-Cre knock-in mice is disrupted by blue light pulse (left traces) but the effect is blocked after application of DHβE (right traces). **(C)** Spiking in two SPNs from ChAT-ChR2-EYFP BAC mice that showed an advanced spike or pause response (left traces) that was blocked after application of DHβE (right traces).

ChI signaling can drive disynaptic inhibition in SPNs by activating nicotinic receptors on nearby terminals (Miura et al., 2006; English et al., 2012; Tritsch et al., 2012; Luo et al., 2013; Nelson et al., 2014b; Faust et al., 2015). We tested whether bath application of the α4β2 nicotinic receptor antagonist, DHβE, would block the response to ChI stimulation that we found in striosomal and matrix SPNs. We recorded from 4 SPNs in the CalDAG-GEFI-GFP-negative (striosomes) and 2 CalDAG-GEFI-GFP-positive (matrix) SPNs, all of which responded with a pause or a spike-advance to the optogenetic stimulation of ChIs (examples shown in Figures 11A,B, left panels). After bath application of DHβE, these same 6 SPNs no longer responded to the light (Figures 11A,B, right panels). Similarly, in ChAT-ChR2-EYFP BAC mice, bath-applied DHβE blocked the light-induced responses of 8/10 SPNs (Figure 11C).

## Discussion

### ChIs send abundant axonal projections into striosomes and matrix circuits implicated in selecting behavioral responses to salient cues

Stereotypy can be viewed as an extreme loss of behavioral flexibility (Graybiel, 2008) and loss of responsiveness to salient cues, a feature recognized in individuals with disorders on the autism spectrum (Gotham et al., 2007). Presentation of an instructive cue induces a semi-synchronized response in striatal ChIs consisting of a brief activation followed by a pause in spiking and then a rebound activation before a return to unsynchronized, tonic firing (Aosaki et al., 1994b; Minamimoto and Kimura, 2002; Goldberg and Reynolds, 2011; Doig et al., 2014; Thorn and Graybiel, 2014). A leading hypothesis is that this ChI response is important for an animal's ability to adapt its behavior in accordance with predicted outcomes (Minamimoto et al., 2005; McCool et al., 2008; Brown et al., 2010; Ding et al., 2010; Goldberg and Reynolds, 2011; Okada et al., 2014). The response of ChIs to cue presentation is dependent upon inputs from the sensory thalamus, the dopaminergic system and the cerebral cortex (Aosaki et al., 1994a; Goldberg and Reynolds, 2011; Doig et al., 2014). Inputs from sensory thalamus to ChI dendrites are matrix-preferring (Fujiyama et al., 2006; Raju et al., 2006). Consistently, we observed that ChI dendrites appeared slightly more prominent in the matrix compartment, relative to the high abundance of ChI axons in both striosomes and matrix. Thus, ChI dendrites, as well as labeling of matrix-preferring inputs from brainstem cholinergic projections (Dautan et al., 2014), likely account for the overall matrix-enriched expression of transgene reporters, immunomarkers and chemical markers for cholinergic processes (Graybiel et al., 1986; Crittenden et al., 2014).

Psychomotor stimulants are well known to induce stereotypic behaviors, and in accordance with this linkage, we found that after repeated high-dose D-amphetamine treatment, male mice were engaged in stereotypy and failed to respond when we introduced the normally salient stimulus of bedding taken from female cages. Multiple brain regions and signaling mechanisms are implicated in evoking stereotypic behaviors of different sorts (Berridge, 2006; Mason and Rushen, 2008). In the dorsal striatum, dopaminergic and cholinergic signaling modulate drug-induced stereotypic behaviors (Capper-Loup et al., 2002; Aliane et al., 2011; Crittenden and Graybiel, 2016). Both increases and decreases in acetylcholine signaling have been implicated in the induction of stereotypic behavior, highlighting how a *balance* of neurotransmitter effects may be what is key to preventing this pathological behavior. For example, the ablation or inhibition of ChI signaling in the striosome-rich anteromedial dorsal striatum prolongs cocaine-induced stereotypy in rats (Aliane et al., 2011), but as well, mice with abnormally high levels of VAChT, thought to augment acetylcholine release, exhibit increased amphetamine-induced stereotypy (Crittenden et al., 2014). Stimulation of the ionotropic nicotinic receptors increases motoric responses to cocaine and amphetamine (Schoffelmeer et al., 2002; Collins and Izenwasser, 2004), whereas antagonism of the slower metabotropic muscarinic signaling system generally increases cocaine-induced responses, including stereotypy (Thomsen et al., 2010; Aliane et al., 2011). Thus, the relationship between cholinergic signaling and stereotypy is complex, with contradictory observations being reported. Some ideas that may help reconcile them are (1) muscarinic and nicotinic transmission may in general have opposite effects, (2) disrupting cholinergic transmission in different districts of the striatum has different effects, and so (3) stereotypies may be controlled both by stimulation and by inhibition of cholinergic transmission, depending on exactly where and how the stimulation or inhibition is accomplished. How this relates to the role of acetylcholine in cue-detection remains unknown.

### Drugs of abuse and imbalanced striosome vs. matrix gene activation

With repeated administration of psychomotor stimulants, overall gene induction in the matrix of the dorsal striatum is suppressed relative to control treatments (Steiner and Gerfen, 1995; Nguyen et al., 2009), and the striosome-to-matrix ratio of c-Fos immediate-early gene induction is increased (Graybiel et al., 1990; Canales and Graybiel, 2000; Vanderschuren et al., 2002; Saka et al., 2004; Jedynak et al., 2012). By contrast, rats engaged in normal behaviors in their home cage are reported to have similar levels of metabolic activity in the two compartments (Brown et al., 2002). Experiments aimed at directly ablating striosomes in rats by intrastriatal injection of MOR1-binding toxins (Tokuno et al., 2002; Lawhorn et al., 2009) have been reported to result in reduced stereotypic responses to methamphetamine and increased c-Fos induction in the matrix (Murray et al., 2014). Our experimental mice showed the expected imbalance in striosome-to-matrix c-Fos induction after a challenge injection of D-amphetamine, with strong nuclear c-Fos immunoreactivity in neurons of GFP-negative striosomes and low levels of c-Fos in neurons of the surrounding matrix. The saline-treated mice showed low-level c-Fos immunostaining that was similar across the compartments. The predominant striosomal activity in drug-treated animals, as measured by the proxy of c-Fos induction, could serve to minimize excess dopamine release driven by drugs of abuse. This speculation would fit with a bare-bones circuit model whereby relative activation of striosomal SPNs directly inhibits dopamine-containing neurons in the substantia nigra pars compacta, and relative silencing of matrix SPNs disinhibits substantia nigra reticulata neurons that thereby are released to inhibit the dopamine-containing neurons.

### ChI modulation of SPN spike firing patterns in striosomal and matrix SPNs

The electrophysiological response of SPNs to direct ChI stimulation has been previously described without classification of striosomal or matrix cell type and thus should favor those in the matrix, which makes up most of the total volume of the striatum. Analyses of SPN responses to ChI stimulation have shown that there are multiple inhibitory post-synaptic currents with fast, slow and very slow time-courses (English et al., 2012), and small excitatory post-synaptic currents (Higley et al., 2011). The inhibitory effects can be transmitted from ChIs to SPNs disynaptically, via nicotinic receptor-mediated release of GABA from neurogliaform and tyrosine hydroxylase-positive striatal interneurons (English et al., 2012; Luo et al., 2013; Faust et al., 2016), from other SPNs (Miura et al., 2006), or from dopamine-containing afferents (Tritsch et al., 2012; Nelson et al., 2014b). Acetylcholine can also inhibit SPNs via muscarinic receptors on SPNs (Clements et al., 2013) and via muscarinic receptor-mediated suppression of glutamate release from cortical terminals (Ding et al., 2010). The effects of ChI activation on striatal microcircuits can be particularly powerful, at least as measured in slice preparations, in that single spikes of ChIs can promote nicotinic receptor-mediated release of GABA and recurrent inhibition (Sullivan et al., 2008; Inoue et al., 2016). The findings raise the possibility that the prominent pause in tonic ChI activity might in fact be preceded by a single, instructive ChI action potential. We found that optogenetic induction of a single spike in ChIs in striatal slices could induce both a brief pause of evoked firing activity and a spike advance in striatal SPNs. Prolonged ChI stimulation appeared to have the same brief consequence on SPN firing patterns.

Relative to SPNs in the matrix, we found that striosomal SPNs showed a greater response to ChI stimulation. Considering the diversity of SPN cell types (Gokce et al., 2016), a larger sample of neurons would make this conclusion firmer. We found that the SPN responses to ChI activation were blocked by bath application of the β2 nicotinic receptor antagonist, DhβE, consistent with a disynaptic inhibition microcircuit, such as that relayed by nicotinic acetylcholine receptors on GABAergic terminals. All together, these results suggest that there may be greater nicotinic receptor-dependent transmission from GABAergic terminals onto at least some striosomal SPNs than onto matrix SPNs. We and others previously found that subsets of nigral neurons (Gerfen et al., 1987; Jimenez-Castellanos and Graybiel, 1987; Prensa and Parent, 2001; Crittenden et al., 2016), which from their positions are likely overlapping with those that synthesize GABA (Kim et al., 2015), appeared to project more abundantly to striosomes than to the matrix. Thus, striosome-enriched terminals from GABAergic dopamine-containing neurons are one potential source of the increased ChI-mediated inhibition of striosomal neurons.

Friedman and colleagues found that relatively selective activation of striosomal SPNs, induced by optogenetic manipulation of prelimbic cortex input fibers, biased rats' cost-benefit trade-off decisions in a T-maze task, so that rats were biased to choose the high-cost/high-value arm over the low-cost/low-value arm (Friedman et al., 2015). This effect appeared to be mediated by a microcircuit in which the activity of high-firing inhibitory interneurons of the striatum was normally enhanced by prelimbic cortical inputs during the trade-off task, leading to suppression of putative striosomal prelimbic-recipient SPNs, but not of non-prelimbic-recipient SPNs of the extrastriosomal matrix. Our results here suggest that other microcircuits, involving ChIs, could be important for the relative inhibition, or disruption of spike timing, in striosomes. It is clearly of great interest that these two classes of striatal interneurons both appear to affect SPN spike timing, a fundamental characteristic of circuit organization and plasticity.

In some instances, and in both compartments, we observed an SPN spike that occurred within 40–50 ms of the light application, and sometimes much sooner, raising the possibility of monosynaptic transmission from ChIs to SPNs. Considering that evidence is sparse for nicotinic acetylcholine receptor mediated currents in SPNs (Luo et al., 2013), these responses could more likely be mediated by glutamate. VGluT3 glutamate transporters are enriched in subsets of ChIs (Gras et al., 2008; Stensrud et al., 2013), and glutamate released from ChIs in the dorsal striatum can induce fast excitatory post-synaptic responses in striatal interneurons (Luo et al., 2013; Nelson et al., 2014a) and in SPNs (Higley et al., 2011).

### Cholinergic signaling in the striosome and matrix compartments is disrupted in slices taken from mice engaged in drug-induced stereotypy

Normally, electrically evoked dopamine release is slightly higher in the matrix than in striosomes (Brimblecombe and Cragg, 2015; Salinas et al., 2016), but in mice in which striosomal neurons are identifiable by Nr4a1-GFP, the ratio is reversed by acute application of cocaine to the brain slices (Salinas et al., 2016). These findings in striatal slices suggest that cocaine disrupts local circuits that maintain the baseline striosome-to-matrix ratio of dopamine signaling. In brain slices from drug-naive rats and mice, neurokinin 1 (NK1) receptor agonists have differential effects on evoked dopamine release in the striosomes and matrix, and these agonist effects are partially affected by cholinergic blockade (Tremblay et al., 1992; Brimblecombe and Cragg, 2015). Part of the effect might also be mediated by somatostatin-containing interneurons (Brimblecombe and Cragg, 2015), which tend to lie near striosomal borders. The possibility that striatal interneurons might mediate direct striosome-matrix communication is further supported by the finding that striatal injection of an NK1 receptor-coupled toxin ablates ChIs and somatostatin-positive interneurons and causes a disruption to the pattern of heightened c-Fos induction in striosomes induced by dopamine agonists (Saka et al., 2004). We have found that ChIs do indeed modulate spike-timing of striosomal and matrix neurons in drug-naive mice and that this modulation is severely blunted in slices taken from mice treated with a schedule of D-amphetamine that induces an imbalance in the striosome-to-matrix ratio of c-Fos expression.

Repeated exposure to psychomotor stimulants strongly impacts ChI signaling in the dorsal striatum (Wang et al., 2013), including partial inhibition of nicotinic acetylcholine receptor mediated dopamine release as measured by fast-scan cyclic voltammetry in slices (Acevedo-Rodriguez et al., 2014). This effect may be a compensatory response to over-stimulation of the dopamine system, considering that ChIs drive nicotinic acetylcholine receptor mediated dopamine release (Zhou et al., 2001; Cachope et al., 2012; Threlfell et al., 2012). Consistently, DHβE application reduces electrically evoked dopamine release in both striosome and matrix compartments (Salinas et al., 2016). We confirmed that optogenetic stimulation induced a spike in ChIs in slices from drug-treated mice, but we did not examine whether baseline ChI firing rates or acetylcholine release were impaired. Thus, whether the ChI-to-SPN signaling defects are pre- or post-synaptic, or both, remains to be tested. Regardless of the mechanism, if semi-synchronized signaling from ChIs to SPNs is important for responsiveness to salient cues, one consequence of drug-repressed cholinergic signaling could be a loss of normal engagement with the environment. When the ChI signaling across striosome and matrix compartments breaks down under drug intoxication, behavioral flexibility and cue responsiveness could be lost.

## Author contributions

CL and FW conducted all recording experiments. JC and CG generated mouse crosses, and conducted behavioral and histology experiments. YL provided reagents. DG performed statistical data analyses. JC, CL, and AG designed experiments, analyzed data and wrote the manuscript.

### Conflict of interest statement

The authors declare that the research was conducted in the absence of any commercial or financial relationships that could be construed as a potential conflict of interest.
